# Fibroblast-Activated Protein Inhibitor PET/CT: Cancer Diagnosis and Management

**DOI:** 10.3389/fonc.2021.758958

**Published:** 2021-11-11

**Authors:** Serkan Kuyumcu, Yasemin Sanli, Rathan M. Subramaniam

**Affiliations:** ^1^ Department of Nuclear Medicine, Istanbul Faculty of Medicine, Istanbul University, Istanbul, Turkey; ^2^ Otago Medical School, University of Otago, Dunedin, New Zealand; ^3^ Department of Radiology, Duke University, Durham, NC, United States

**Keywords:** PET, cancer-associated fibroblast, fibroblast activation protein, theranostic, 68Ga-FAPI04, FAPI

## Abstract

Fibroblast activation protein (FAP), overexpressed on cancer-associated fibroblasts (CAFs), is a novel target for molecular imaging of various tumors. Recently, the development of several small-molecule FAP inhibitors for radiolabeling with ^68^Ga has resulted in the emergence of studies evaluating its clinical role in cancer imaging. Preliminary findings have demonstrated that, in contrast to radiotracers taking advantage of cancer-specific targets such as PSMA and DOTATATE, FAPs as a target are the most promising that can compete with ^18^FDG in terms of widespread indications. They also have the potential to overcome the shortcomings of ^18^FDG, particularly false-positive uptake due to inflammatory or infectious processes, low sensitivity in certain cancer types, and radiotherapy planning. In addition, the attractive theranostic properties may facilitate the treatment of many refractory cancers. This review summarizes the current FAP variants and related clinical studies, focusing on radiopharmacy, dosimetry, and diagnostic and theranostic applications.

## Introduction

Historically, cancer imaging has focused on morphological anatomy, as opposed to molecular imaging, which targets physiological activity in a specific tissue by utilizing modalities that use certain probes to overcome the poorly reflected biology of cancer by anatomical imaging. In this regard, the association between glycolysis and cancer cell metabolism has well been translated into PET imaging in cancer, and ^18^F-FDG PET/CT has revolutionized cancer imaging and gained widespread acceptance for managing various malignancies. More than 40 years of success of ^18^F-FDG has also led to the successful integration of specific radiotracers such as ^68^Ga-labeled somatostatin analogs and prostate-specific membrane antigens (PSMA) into clinical practice (1) in the last decade, resulting in an exponentially decreasing timeframe for widespread acceptance. Similarly, the recent emergence of radiolabeled fibroblast activation protein (FAP) inhibitors (FAPI) with pan-cancer targeting features hints at relatively rapid adoption. FAPs are overexpressed by cancer-associated fibroblasts (CAFs) found in the tumor stroma of various cancers, and several radiolabeled FAPI variants have already been introduced as promising targets for PET/CT imaging (2–6). The excellent imaging contrast, low activity in normal organs, and theranostic potential are encouraging; however, activated fibroblasts in benign conditions with inflammatory and wound-healing processes can also express FAPs. This review summarizes the pathophysiology of FAPs and clinical implications of FAP-targeted PET/CT data in malignant diseases focusing on radiopharmaceuticals and dosimetry. In this regard, a search of PubMed, MEDLINE, and Scopus databases with one or more combinations of the following terms: “FAPI”, “FAPI PET”, “cancer-associated fibroblast”, “fibroblast activation protein”, and “fibroblast activation protein inhibitor” was performed. All papers in English were evaluated and were included if they fell within the scope of this review.

## Physiology and Pathology

FAP is a type II transmembrane serine protease expressed in activated tumor stroma and inflamed tissues during wound healing (7). Overexpression has been seen in most epithelial cancers, especially in tumors with a high degree of desmoplasia (8). FAP is not overexpressed by tumor cells themselves; it is overexpressed by CAFs that are responsible for tumor growth, aggressiveness, and migration, which consist of a high tumor volume within the tumor stroma (9); thus, a high expression of FAP on CAFs may be considered a factor of aggressiveness of tumor behavior and poor prognostication (10). The tumor microenvironment (TME) plays a crucial role in the survival, proliferation, and spread of tumor cells (11). FAP is overexpressed on the cancer-related fibroblast cell membrane and TME stromal cells. On the other hand, FAP is barely expressed in healthy adult tissues, except uterine stroma, particularly in the proliferative phase, pancreatic alpha cells, human placenta, and some dermal fibroblasts (12). Because of minimal expression in normal tissue, labeled FAP *via* radiopharmaceuticals is seen as a promising target in diagnosis as well as therapy in oncology (13).

## Radiopharmaceuticals and Dosimetry

It has been over 30 years since Garin‐Chesa et al. proposed the surface glycoprotein of reactive stromal fibroblasts as potential antibody targets in human epithelial cancer (14). Consecutive attempts for imaging FAPs (15) acknowledged the disadvantages and challenges of the initial compounds, leading to the introduction of small-molecule FAP inhibitors with more favorable characteristics, particularly increased selectivity and affinity (16, 17). The preliminary human applications using the early FAP inhibitor, namely FAPI02, demonstrated high tumor specificity but declining uptake over time. Consequently, Lindner et al. (18) evaluated a group of novel tracers derived from FAPI02 to improve tumor uptake and retention and accordingly proposed FAPI04 as a more suitable tracer with the potential for theranostic applications. A dosimetry study including 50 patients with various cancers by Giesel et al. also confirmed the higher tumor retention time of ^68^Ga‐FAPI04 than ^68^Ga‐FAPI02. The estimated effective doses for ^68^Ga‐FAPI04 and ^68^Ga‐FAPI02 PET/CT were reported as 1.80E−2 mSv/MBq and 1.64E−2 mSv/MBq, respectively, which is similar to that of clinically established PET imaging procedures. These values are comparable or lower than the effective dose of PET/CT imaging with ^18^F-FDG (1.9E−2 mSv/MBq) (19), ^68^Ga-labeled somatostatin analogs (2.1E−2 mSv/MBq) (20), and ^68^Ga-PSMA (1.71E−2–2.3E−2 mSv/MBq) (21).

Further research on FAPI molecules has been conducted to improve the therapeutic efficacy through higher-dose delivery. Higher tumor-to-organ/blood ratios achieved with FAPI21 and FAPI46, as reported by Loktev et al. (3), were promising; however, due to increased uptake of FAPI21 in the oral mucosa, thyroid, and salivary glands, FAPI46 was presumed more favorable. Accordingly, clinical imaging studies with ^68^Ga-FAPI46 in a cohort of 69 patients by Ferdinandus et al. (22) and six patients by Koerber et al. (20) have demonstrated encouraging results. Another novel FAP inhibitor with a different structure based on the squaric acid motif, DOTA.SA.FAPi has also been introduced (23). Human studies also confirmed high target-to-background ratios achieved with colorectal cancer xenograft mouse model in a cohort of 54 patients (24). The mean effective dose equivalent was 1.64E−2 mSv/MBq, similar to other FAPI PET studies.

Most FAP inhibitors have been labeled using the DOTA derivatives; nevertheless, NOTA chelators have also been studied. A FAP inhibitor, FAPI74, which allows labeling with ^18^F and ^68^Ga, has also been studied in a cohort of 10 patients (4). High contrast imaging and low radiation burden using ^18^F-FAPI74 were reported (effective dose rate of 1.4E−2 mSv/MBq). Wang et al. (6) also introduced a NOTA-FAPI, Al^18^F-NOTA-FAPI, with comparable affinity with several other FAPI probes. They reported successful imaging of 10 cancer patients and calculated the whole-body effective dose of 1.24E−02 mSv/MBq. Another FAPI molecule that allows ^18^F labeling is the glycosylated FAP inhibitor (^18^FFGlc-FAPI). Toms et al. (25) evaluated ^18^FFGlc-FAPI in the preclinical setting and proposed it as a candidate that can take advantage of extended PET imaging provided by the longer physical half-life of ^18^F and higher tumor retention of Glc-FAPI. The only non-PET radiotracer is reported by Linder et al. (23). The authors have studied novel FAPI variants for labeling using the theranostic pair, ^99m^Tc, and ^188^Re. FAPI34 was labeled with ^99m^Tc, and SPECT scans of two patients were comparable with PET imaging with ^68^Ga-FAPI46.

Despite the enthusiasm that FAPI agents have gained in cancer imaging, the experience on targeted radionuclide applications is mainly restricted to a small number of cases. Thus, data on effective dose rates for therapeutic radionuclides is far limited. In the clinical setting, two metastatic breast cancer patients tolerated treatments very well with ^177^Lu-DOTA.SA.FAPi ^(^ 26) and ^90^Y-FAPI04^18^, and preliminary results indicated that the treatment was safe. Linder et al. (23) and Kratochwil et al. (27) have treated two patients with ^90^Y-FAPI46 and one with ^153^Sm-FAPI46; however, no dosimetric results were reported. Despite the preliminary patient reports on FAP-targeted radionuclide treatments, the data on dosimetry of normal organs is still lacking. Recently, Kuyumcu et al. (28) reported estimated radiation-absorbed doses to normal organs using low-dose ^177^Lu-FAPI04. The estimated radiation dose to critical organs was significantly low compared with clinically established targeted radionuclide therapies, particularly ^177^Lu-DOTATATE and ^177^Lu-PSMA. Bone marrow was the dose-limiting organ, and the authors concluded that up to 50 GBq of cumulative activity could be tolerated. These results are expected regarding the high image contrast; however, the relatively short tumor retention time requires dose increase to achieve tumoricidal effects. Therefore, further research is necessary to optimize the therapeutic efficacy and determine the safety of high-dose radionuclide treatments. Baum et al. (29) reported comparable estimated radiation dose to critical organs using ^177^Lu-labeled FAP-2286; however, a higher radiation dose to tumoral lesions was achieved, justifying further investigation.

## Fibroblast Activation Protein-Targeted Imaging in Oncology

Early clinical trials have evaluated patient groups with various cancers. In 2018, the first PET imaging of FAPs in three patients was reported by Loktev et al. (30) as a proof-of-concept study. Tracer uptake with a high tumor-to-background ratio was noted in breast, lung, and pancreatic cancers. Consequently, Kratochwil et al. (31) from the same team reported the remarkable ^68^Ga-FAPI PET/CT results of 80 cancer patients with 28 different tumor types. The uptake values highly varied between different tumor types as well as individuals. The highest uptake of ^68^Ga-FAPI (SUV_max_ >12) was detected in sarcoma, esophageal, breast, cholangiocarcinoma, and lung cancer patients, while pheochromocytoma, renal cell, differentiated thyroid, and gastric cancers were the lowest (SUV_max_ <6). The low background activity resulted in excellent image contrast despite the intratumoral and interindividual variability even with low tumor activity. In a preliminary study, Giesel et al. (2) evaluated different FAPI variants in a cohort of 50 cancer patients. Similarly, high ^68^Ga-FAPI uptake was observed in esophageal, pancreatic, head and neck, nonsmall cell lung, and colon cancers in contrast to low or no uptake in dedifferentiated thyroid cancer. The authors have demonstrated the first comparative evaluation of ^68^Ga-FAPI PET/CT imaging with ^18^FDG PET/CT in six patients as distinct from other preliminary studies.

Chen et al. (32) compared ^68^Ga-FAPI04 PET/CT with ^18^FDG PET/CT in a larger cohort of 75 patients. ^68^Ga-FAPI04 PET/CT was superior to ^18^FDG PET/CT in newly diagnosed 54 patients with 12 tumor types. Similarly, ^68^Ga-FAPI04 was superior in 21 patients with eight cancer types who underwent PET/CT for restaging. The uptake of ^68^Ga-FAPI04 was significantly higher and resulted in high contrast images with the highest uptake in sarcoma, pancreatic, liver, and esophageal cancers. Ten patients with high FAPI04 uptake were negative on ^18^FDG PET/CT, particularly hepatocellular, gastric, and pancreatic cancers. The sensitivity of ^68^Ga-FAPI04 PET/CT was significantly higher than ^18^FDG in detecting bone, visceral, and lymph node metastases; however, the specificity was lower. Therefore, false positivity also applies to ^68^Ga-FAPI04 as a potential diagnostic pitfall and requires careful evaluation. However, ^68^Ga-FAPI04 PET/CT outperformed ^18^FDG PET/CT in patients with liver metastasis and peritoneal carcinomatosis. Another comparative study by Chen et al. (33) evaluated 68 cancer patients with inconclusive ^18^FDG-PET/CT findings. Fifty-nine of the patients had histopathologically confirmed malignant disease, and most of the FDG-negative or inconclusive patients, mainly gastric and liver cancers, presented significantly increased 68Ga-FAPI04 uptake. Higher uptake was also noted with peritoneal carcinomatosis, liver, and skeletal metastases. On the other hand, despite high ^18^FDG uptake in the metastatic brain lesions, the tumor-to-background ratio on ^68^Ga-FAPI04 PET/CT was higher due to lack of background activity. The authors have highlighted the complementary role of ^68^Ga-FAPI04 imaging in patients with inconclusive ^18^FDG PET/CT findings.

The encouraging results of studies investigating various cancers have led to the emergence of ^68^Ga-FAPI PET studies in specific cancer types. Head and neck cancers are among the most investigated cancers as a target of FAP-directed PET imaging. In a cohort of 45 patients with nasopharyngeal cancers, ^68^Ga-FAPI04 PET/CT was superior to ^18^FDG PET/CT in detecting primary tumors, lymph nodes, and metastatic disease, resulting in management changes in 18% of the patients (34). Qin et al. (35) compared ^68^Ga-FAPI04 with ^18^FDG PET/MR in 15 patients with nasopharyngeal carcinoma. ^68^Ga-FAPI04 uptake in the primary tumors was lower than ^18^FDG uptake, although not statistically different. On the other hand, lower ^68^Ga-FAPI04 uptake in the metastatic lymph nodes was statistically significant; however, ^68^Ga-FAPI04 imaging outperformed ^18^FDG in detecting unknown distant metastases and improved primary tumor delineation for differentiation of skull-base and intracranial invasion. Another study (36) evaluated 14 patients with head and neck cancers and compared ^68^Ga-FAPI04 PET/CT with ^18^FDG-PET/CT for differentiating between healthy and tumor tissue. In a cohort of 12 patients with adenoid cystic carcinomas, Röhrich et al. (37) reported that ^68^Ga-FAPI PET/CT increased staging accuracy. ^68^Ga-FAPI04 PET/CT was also proposed as a feasible imaging method in 10 patients with oral squamous cell carcinoma by Linz et al. (38), although they did not reach a firm conclusion.

Another cancer group of interest for FAP-targeted imaging is the gastrointestinal malignancies. The advantages of ^68^Ga-FAPI04 over ^18^F-FDG PET/CT in the imaging of esophageal cancer were reported as single-case studies (39, 40) and two small cohort studies which investigated the potential of ^68^Ga-FAPI04 PET/CT on target volume delineation for radiotherapy planning. Overexpression of FAPs in gastric carcinomas has also been demonstrated (41–43). Quin et al. investigated 20 gastric cancer patients and described the superiority of ^68^Ga-FAPI04 PET/MR over ^18^F-FDG PET/CT in visualizing the primary tumors and most metastatic lesions (44). Pang et al. (45) evaluated ^68^Ga-FAPI04 PET/CT of 20 patients with gastric carcinoma and reported higher detection rates and mean SUV_max_ than 18FDG PET/CT. The authors have also evaluated patients with duodenal (*n* = 2) and colorectal cancers (*n* = 13). The duodenal adenocarcinomas were ^18^FDG negative and demonstrated ^68^Ga-FAPI04 uptake. On the other hand, ^68^Ga-FAPI04 and ^18^FDG PET/CT detected all primary lesions in colorectal cancer patients; however, significantly higher SUV_max_ and higher tumor-to-background contrast resulted in more precise tumor delineation. In an investigation into lower gastrointestinal tract tumors, Koerber et al. (20) evaluated the role of FAPI PET/CT in colon, sigmoid, rectal, and anal cancers. The authors concluded that primary and metastatic tumors could be accurately detected by ^68^Ga-FAPI PET/CT changing TNM status and disease management. Peritoneal carcinomatosis patients (*n* = 46) were evaluated by Zhao et al. (46), and the authors reported sensitivity of ^68^Ga-FAPI04 PET/CT superior to ^18^F-FDG PET/CT in detecting diffuse or nodular type disease. They also noted that significantly higher tracer uptake was mainly observed in peritoneal carcinomatosis from gastric cancer.

The characteristics of FDG uptake in primary liver malignancies are unpredictable, particularly in hepatocellular carcinoma (HCC) due to factors such as low metabolism and physiological liver activity (47). In a cohort of 17 patients, Shi et al. reported higher ^68^Ga-FAPI04 uptake in malignant liver nodules than in benign nodules (48). The authors have also evaluated hepatocellular carcinoma (*n* = 14) and intrahepatic cholangiocarcinoma (*n* = 3) patients in another study (49) and concluded the superiority of ^68^Ga-FAPI04 PET/CT over ^18^F-FDG PET/CT in the detection of primary hepatic malignancies. Guo et al. (50) confirmed the superiority of ^68^Ga-FAPI04 PET/CT in 20 patients with hepatocellular carcinoma and 12 patients with intracellular cholangiocarcinoma with a sensitivity equivalent to that of contrast-enhanced CT and MRI. They also reported two patients with benign nodules that were ^68^Ga-FAPI negative and highlighted its potential in differentiating benign from malignant lesions. Similarly, the ability of dynamic ^68^Ga-FAPI04 PET/CT in differentiating HCC from non-HCC lesions has also been demonstrated in a brief report (51). ^68^Ga-FAPI04 PET/CT was compared with contrast-enhanced CT in 19 pancreatic ductal adenocarcinoma patients, and ^68^Ga-FAPI04 PET/CT results changed TNM staging in 10 patients. However, the authors have noted challenges of differentiating pancreatitis from adenocarcinoma (52). In another study, Liermann et al. (53) compared ^68^Ga-FAPI04 PET/CT with ceCT in seven recurrent pancreatic patients for radiotherapy planning. However, both studies did not use ^18^F-FDG PET/CT for comparison.

In a study by Komek et al. (54), the authors compared the ^68^Ga-FAPI04 with ^18^F-FDG PET/CT of 20 breast cancer patients and concluded that ^68^Ga-FAPI04 was superior to ^18^F-FDG in detecting the primary tumor and the metastatic lesions with high sensitivity and tumor-to-background ratio. In a study by Dendl et al. (55), investigating patients with various gynecological malignancies, 14 patients had breast cancer, and the authors have reported strong to moderate FAP expression in the stroma of breast carcinomas. FAP expression in ovarian (*n* = 9), cervical (*n* = 4), endometrial (*n* = 2), and tubal cancers (*n* = 1) in addition to one patient with uterine leiomyosarcoma was also investigated. High tracer uptake and low background activity in gynecological tumors resulted in excellent image contrast compared with ^18^F-FDG, and the authors recommended further research on clinical applications.

Koerber et al. (56) evaluated the role of ^68^Ga-FAPI imaging in a cohort of 15 patients diagnosed with various sarcoma subtypes. The excellent tumor-to-background ratio was achieved in primary tumors and metastases, including low-grade sarcomas, where ^18^F-FDG PET/CT has limitations. Accordingly, ^68^Ga-FAPI PET/CT was highlighted as a promising probe with the potential for the theranostic approach. Kessler et al. (57) evaluated 47 patients with bone or soft tissue sarcomas and measured a significant association between tracer uptake and histopathological FAP expression. High sensitivity and PPV of FAPI PET resulted in upstaging in eight (13%) patients and management change in 13 (30%) patients compared with FDG-PET.

^18^F-FDG PET/CT has a limited role in diagnosing malignant brain tumors and is particularly useful in distinguishing recurrent tumors from radiation necrosis. On the other hand, lack of background activity in FAP-targeted imaging provides high image contrast, and this advantage over FDG PET has been addressed in various reports, particularly for brain metastases. Regarding primary brain tumors, two studies have evaluated FAP-targeted imaging for glioblastomas. Windisch et al. (58) has studied 14 glioblastoma patients in the setting of radiotherapy planning. A diagnostic study by Rohrich et al. (59) in 18 glioma patients evaluated FAP-specific imaging as a promising new tool to differentiate between low-grade and high-grade diseases. In this regard, PET imaging of FAPs may potentially be used as a noninvasive probe for predicting malignant progression of IDH-mutant WHO grade II gliomas to grades III and IV over time, which may have severe therapeutic consequences.

^18^F-FDG PET/CT has a well-established impact on high-grade lymphoma management; however, its role in indolent, low-grade disease is controversial. Recently, Jin et al. (60) investigated 11 Hodgkin lymphoma and 62 non-Hodgkin lymphoma patients, and increased radiotracer uptake was observed in Hodgkin lymphoma. Indolent lymphomas showed mild uptake in contrast to aggressive non-Hodgkin lymphomas with high uptake, which resulted in a positive association between the corresponding clinical classification of non-Hodgkin lymphomas.

**Figure 1:**
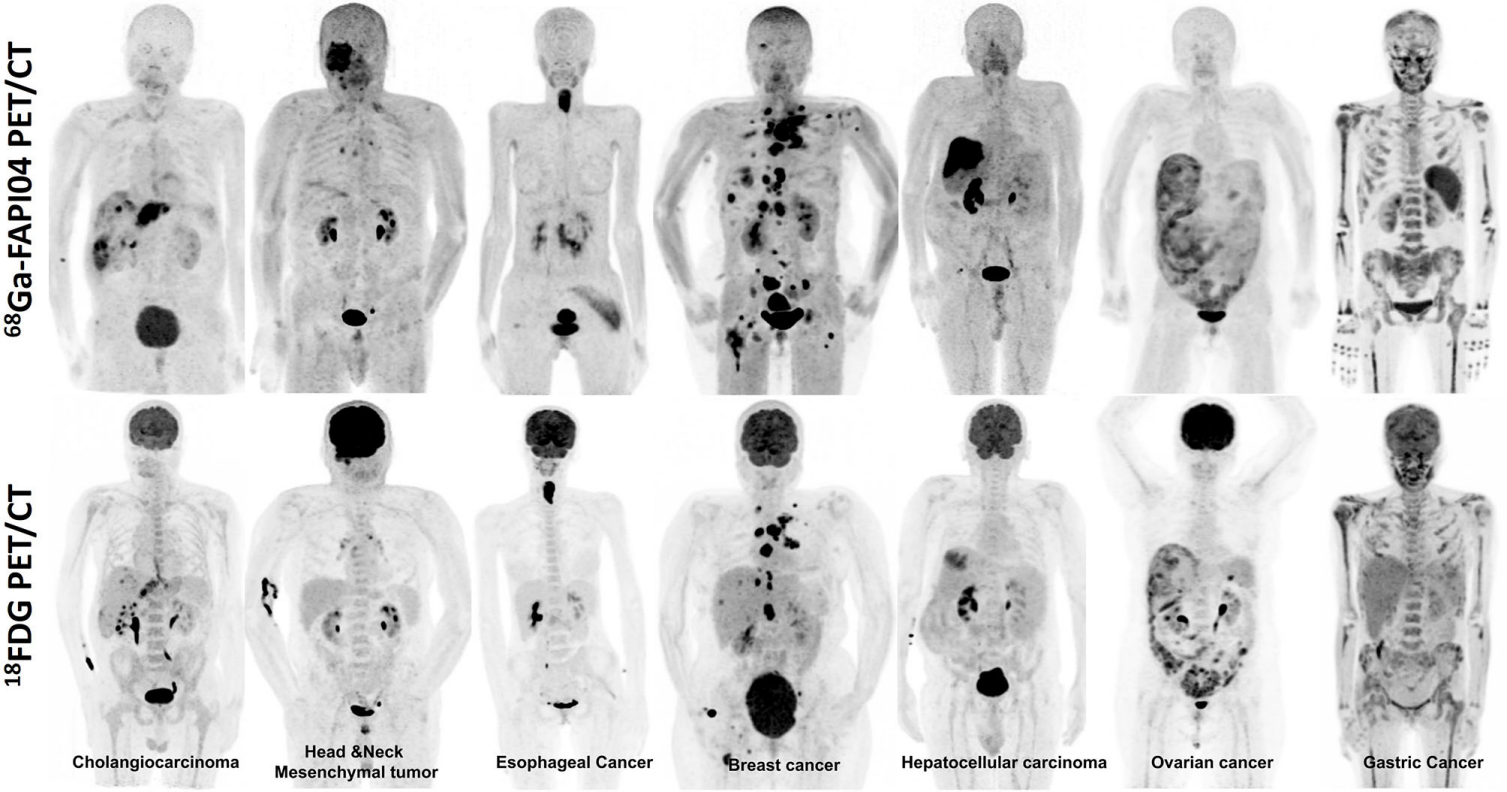
Intraindividual comparison of ^18^F-FDG and ^68^Ga-FAPI04 PET maximum-intensity projection images of seven patients with various histopathologically proven tumors. The uptake of ^68^Ga-FAPI04 is superior or equal to ^18^F-FDG in the metastatic lesions.

In a recent meta-analysis, Sollini et al. (61) evaluated 23 studies that included 17 oncologic and six non-oncologic articles to evaluate the potential role of ^68^Ga-FAPI imaging. They found that the superiority of ^68^Ga-FAPI over ^18^F-FDG was observed especially in abdominal cancers in detecting either the primary tumor or the nodal and distant metastases. They demonstrated estimated pooled sensitivity and specificity of patient-based ^68^Ga-FAPI imaging were 0.99 (95% CI, 0.97–1.00; *I* ^2^ = 0.00%; *p* = 0.75) and 0.87 (95% CI, 0.62–1.00; *I* ^2^ = 0.00%; *p* = 0.51) with negligible heterogeneity, respectively. On the contrary, the lesion-based analysis revealed high heterogeneity in sensitivity and specificity. Meanwhile, pooled sensitivity for the primary tumor and distant metastases was found 1.00 (95% CI, 0.98–1.00; *I* ^2^ = 0.00%; *p* = 0.51) and 0.93 (95% CI, 0.88–0.97; *I* ^2^ = 0.00%; *p* = 0.41) with negligible heterogeneity, respectively, whereas pooled sensitivity and specificity of nodal metastases had high heterogeneity (*I* ^2^ = 89.18% and *I* ^2^ = 95.74). Consequently, FAPI PET was demonstrated as a promising radiopharmaceutical, especially in some malignancies with low FDG uptake in the primary tumor or its metastasis. **Table 1** summarizes the studies evaluating FAP-targeted imaging and **Figure 1** illustrates intraindividual comparison of ^18^FDG and ^68^Ga-FAPI04 PET/CT in various cancer entities.

**Table 1:** Summary of studies evaluating FAP-targeted imaging of various cancer types.

Reference	Malignant disease	*n*	Study outcome
Loktev et al. (30)	Various types of cancer	8	FAPI allows rapid and quality imaging and labeling with therapeutic isotopes in tumors with high stromal content.
Kratochwil et al. (31)	Various types of cancer	80	The highly selective tumor uptake of FAPI may reveal new applications for noninvasive tumor characterization, staging imaging, or radioligand therapy.
Giesel et al. (2)	Various types of cancer	50	FAPI-targeted PET does not require any diet or fasting; it has better image quality than ^18^F-FDG, and acquisition can be obtained earlier.
Chen et al. (32)	Various types of cancer	75	^68^Ga-FAPI PET/CT showed superior diagnostic efficiency compared with 18F-FDG PET/CT, particularly in the diagnosis of liver metastases, peritoneal carcinomatosis, and brain tumors.
Chen et al. (33)	Various types of cancer	68	In patients with inconclusive ^18^F-FDG PET/CT findings, ^68^Ga-FAPI may have a complementary role in differentiating malignant lesions, locating the primary site of unknown malignancy.
Zhao et al. (34)	Nasopharyngeal carcinoma	45	^68^Ga-FAPI PET/CT can diagnose primary and metastatic nasopharyngeal carcinoma and supplement MRI for T staging and radiotherapy planning.
Qin et al. (35)	Nasopharyngeal carcinoma	15	^68^Ga-FAPI shows better diagnostic performance than ^18^F-FDG in patients with nasopharyngeal carcinoma.
Syed et al. (36)	Head and neck cancer	14	A novel approach of tumor detection, contouring, and targeted radiotherapy of head and neck cancers using ^68^Ga-FAPI PET.
Röhrich et al. (37)	Adenoid cystic carcinoma	12	^68^Ga-FAPI PET/CT is a promising imaging modality for adenoid cystic carcinomas, increasing the accuracy of staging exams and radiotherapy planning volumes compared with conventional CT and MRI.
Linz et al. (38)	Oral cavity squamous cell carcinoma	10	Compared with ^18^F-FDG and cervical MRI, ^68^Ga-FAPI can reduce patient morbidity, minimizing the number of neck dissections due to false-positive cervical lymph nodes.
Qin et al. (44)	Gastric carcinomas	20	^68^Ga-FAPI PET/MR showed better diagnostic performance than ^18^F-FDG PET/CT in visualizing the primary and metastatic lesions of gastric cancer.
Pang et al. (45)	Gastric, duodenal, and colorectal cancers	35	^68^Ga-FAPI PET/CT showed superior diagnostic performance. To compare the diagnostic efficacy of 68Ga FAPI PET/CT and ^18^F-FDG PET/CT in primary and metastatic cancers of the gastrointestinal tract.
Koerber et al. (20)	Colon, sigmoid, rectal, and anal cancers	22	^68^Ga-FAPI PET/CT imaging of lower gastrointestinal tract primary and metastatic tumors resulted in changes in TNM staging and treatment management.
Zhao et al. (46)	Peritoneal carcinomatosis	46	^68^Ga-FAPI04 PET/CT showed superior diagnostic performance compared with ^18^F-FDG PET/CT to detect peritoneal carcinomatosis, particularly in gastric cancer.
Shi et al. (48)	Liver malignancies	17	^68^Ga-FAPI04 has demonstrated high sensitivity, particularly in the detection of poorly differentiated hepatic malignancies.
Shi et al. (49)	Liver malignancies	20	^68^Ga-FAPI PET/CT has superior potential for imaging of hepatic tumors compared with ^18^F-FDG PET/CT.
Guo et al. (50)	Liver malignancies	34	The sensitivity of ^68^Ga-FAPI04 PET/CT in detecting primary and metastatic liver lesions is equivalent to that of contrast-enhanced CT and MRI and better than ^18^F-FDG PET/CT.
Geist et al. (51)	Hepatic lesions	8	Dynamic ^68^Ga-FAPI PET scan allows differentiation between hepatocellular carcinoma and non-HCC lesions
Röhrich et al. (52)	Pancreatic ductal adenocarcinomas	19	^68^Ga-FAPI PET/CT allowed restaging in half of the patients with PDAC and the majority of patients with recurrent disease compared with standard-of-care imaging.
Liermann et al. (53)	Pancreatic cancer	7	FAPI PET/CT seems to be a superior imaging modality to contrast-enhanced CT, which is the current gold standard in pancreatic cancer with the potential as a tool for automatic target volume definition before radiotherapy.
Kömek et al. (54)	Breast cancer	20	^68^Ga-FAPI04 PET/CT is superior to 18F-FDG PET/CT in detecting primary tumors and metastases in breast cancer patients.
Dendl et al. (55)	Gynecological malignancies	31	^68^Ga FAPI PET/CT seems to be a more promising imaging modality for staging and follow-up of gynecological tumors compared with ^18^F-FDG PET/CT
Koerber et al. (56)	Several types of sarcoma	15	^68^Ga-FAPI PET/CT has a high potential for clinical use in patients diagnosed with sarcoma as a staging probe and tumor characterization.
Kessler et al. (57)	Bone and soft tissue sarcomas	47	There is a correlation between tumoral FAPI uptake intensity and histopathological FAP expression in sarcoma patients, and FAPI PET has a high sensitivity.
Windish et al. (58)	Glioblastoma	14	For target volume delineation, ^68^Ga-FAPI PET outperformed MRI in detecting gross tumor volumes.
Röhrich et al. (59)	Gliomas	18	Using FAP-specific PET imaging may allow a noninvasive distinction between low-grade IDH-mutant and high-grade gliomas.
Jin et al. (60)	Different subtypes of lymphomas	73	^68^Ga-FAPI imaging may be an alternative method for detecting FAP expression in lymphoma lesions and characterizing lymphoma profiles.
Sollini et al. (61)	Various types of cancer	482	68Ga-FAPI PET appears to be a suitable method for the detection of primary lesions and distant metastases of malignancies that are not particularly suitable for 18F-FDG PET imaging.

## Radiotherapy Planning

Radiotherapy can be used as neoadjuvant or adjuvant therapy in many tumors. Accurate tumor delineation is the most critical part of therapy planning because it directly affects therapy response. Although computed tomography (CT) is the most commonly used modality, numerous studies in literature showed that 18F-FDG PET/CT could be used for delineating metabolic tumor volume to predict tumor response and tumor delineation for radiotherapy planning (62, 63). On the other hand, there is no consensus on the optimal method with ^18^F-FDG PET/CT imaging because of false-positivity/negativity-like infection/inflammation or masking of the FDG uptake due to tumor location. For this reason, FAPI PET/CT can be a useful alternative radiopharmaceutical to ^18^F-FDG in radiotherapy planning.

Currently, a limited number of articles are available related to radiotherapy planning in the literature. In one of these, Zhao et al. compared the usefulness of ^68^Ga-FAPI with ^18^F-FDG PET/CT imaging in evaluating gross tumor volume (GTV) delineation in 21 locally advanced esophageal cancer patients (64). They showed that ^68^Ga-FAPI had significantly higher radiotracer uptake than ^18^F-FDG, especially when the primary tumor was in the middle or lower thoracic esophagus. Moreover, the authors showed that ^68^Ga-FAPI PET/CT had a higher success rate than ^18^F-FDG PET/CT for detecting metastatic lymph nodes. In addition to this study, Ristau et al. also evaluated the impact of primary staging with ^68^Ga-FAPI PET/CT on radiotherapy planning in esophageal cancer patients (65). They showed that primary tumors demonstrated high FAPI uptake with excellent tumor-to-background ratios that resulted in accurate target volume delineation.

Syed et al. compared GTV between ^68^Ga-FAPI PET/CT and conventional radiologic imaging methods such as contrast-enhanced CT and MRI in head and neck cancer in 14 patients (36). Of these, two patients had taken additive radiotherapy after surgical resection for macroscopic residual tumors, whereas the rest of the 12 patients received radiotherapy in the definitive setting with a prior biopsy for histopathological confirmation. They have used four different thresholds (three-, five-, seven-, and tenfold increased uptake) of FAPI uptake in the primary tumor and normal tissue. Eventually, the authors showed that primary tumors had high FAPI avidity, while low background uptakes were shown in healthy tissues in the head and neck region. GTV was found to have significant disparities between all threshold levels of FAPI-GTV to CT-GTV. Also, Röhrich et al. compared the clinical potential of conventional imaging and ^68^Ga-FAPI PET/CT for staging and radiotherapy planning in a total of 12 (seven primary, five recurrent) adenoid cystic carcinoma patients in the head and neck region (37). They demonstrated that ^68^Ga-FAPI PET/CT led to upstaging in two of 12 patients and to the detection of additional metastases in three patients and thus staging was altered in 42% of patients with ^68^Ga-FAPI PET/CT. Moreover, they showed that when compared with conventional imaging, the accuracy of target volume delineation for radiotherapy improved with FAPI PET. In addition, in a pilot study, 13 glioblastoma patients who were candidates for radiotherapy were evaluated with FAPI PET compared with MRI (58). FAP-specific GTV was created using a five-, seven-, and tenfold threshold of increased uptake compared with normal tissue, and MRI-specific GTV was created based on T1-weighted images. They demonstrated that FAP-specific GTV were significantly different from the MRI-GTV for FAP fivefold threshold but not with FAP seven- and tenfold thresholds. FAP-specific PET target volume delineation was not found covered by MRI-GTVs in this study. All of these studies need to be supported by further studies with larger sample sizes.

## Theranostics

FAP-targeted diagnostic imaging has so far shown promising potential for a broad spectrum of cancers. However, taking theranostic properties and low tracer uptake in nontarget organs into account, the possibility of radionuclide treatments of cancers that are currently not in the scope of nuclear medicine is most appealing. The knowledge of theranostic applications using therapeutic radionuclides such as ^177^Lu, ^90^Y, and ^225^Ac in metastatic neuroendocrine and prostate cancers will likely accelerate new research data on FAP-targeted radionuclide treatments. Still, the therapeutic applications are limited. The first reported FAP-targeted radionuclide treatment using ^90^Y-FAPI04 was administered to a metastatic breast cancer patient by Lindner et al. (18). The posttreatment bremsstrahlung images were in line with ^68^Ga-FAPI04 PET images, and the treatment was well tolerated with no adverse effects observed. A significant reduction in pain medication proved the potential efficacy of the treatment. Dendl et al. (55) reported temporary stable disease in patients with metastatic breast and colon cancer after receiving four cycles of ^90^Y-FAPI04 treatment. Another progressive metastatic breast cancer patient was reported by Ballal et al. (26). The authors used a novel FAP agent based on the squaric acid motif with improved structural features and administered the patient with 3.2 GBq of ^177^Lu-DOTA.SA.FAPi on compassionate grounds. The primary tumor and the metastatic brain lesion received 1.48E mGy/MBq and 3.46 mGy/MBq absorbed dose. Jokar et al. (66) also reported a metastatic breast carcinoma patient who had failed conventional treatments and received two cycles of ^177^Lu-FAPI46. Kratochwil et al. (27) treated a metastatic sarcoma patient using ^153^Sm and ^90^Y, reaching a cumulative dose of 20 GBq ^153^Sm-FAPI46 and 8GBq ^90^Y-FAPI46 in three cycles. The authors reported 8 months of stable disease, encouraging further studies. So far, two comprehensive studies of FAP-targeting radionuclide treatment have been reported. Baum et al. (29) studied FAP-2286 and administered ^177^Lu-labeled FAP-2286 to 10 patients with pancreas, breast, ovarian, and rectal cancers. Although a treatment response was not achieved, the authors concluded the reasonable toxicity profile with well-tolerated adverse effects. Assadi et al. (67) have administered ^177^Lu-FAPI46 to 21 patients with various cancers and reported stable disease in 12 of the patients, emphasizing the tolerability and safety of the treatment. The results of both studies agreed that current results warranted further investigation. In summary, the preliminary studies (**Table 2**) have reported low estimated radiation dose to nontarget organs compared with well-established radionuclide therapies such as PSMA and DOTATATE. The tumor retention time of FAP inhibitor compounds has evolved since their first introduction; however, dose-escalation studies to achieve tumoricidal effects and optimize the therapeutic efficacy for different tumors require further research.

**Table 2:** Summary of studies evaluating FAP-targeted radionuclide treatments.

Reference	Malignancy	*n*	Radiopharmaceutical	Administered dose	Tumor absorbed dose	Cycles	Response
Lindner et al. (18)	Breast	1	^90^Y-FAPI04	2.9 GBq	n/a	1	Reduction in pain medication
Ballal et al. (26)	Breast	1	^177^Lu-DOTA.SA.FAPi	3.2 GBq	1.48 and 3.46 mGy/MBq	1	Decrease in the intensity of headaches. No adverse effects
Kratochwil et al. (27)	Sarcoma	1	^153^Sm-FAPI46 and ^90^Y-FAPI46	20 GBq ^153^Sm- and 8 GBq ^90^Y-FAPI46 (*cumulative*)	n/a	3	Stable disease for 8 months
Baum et al. (29)	Various	10	^177^Lu-FAP-2286	5.8 ± 2.0 GBq (*mean*)	3 ± 2.7 Gy/GBq (*mean*)	2 (*mean*)	PD (*n* = 10), SD (*n* = 1); well tolerated, no adverse symptoms
Dendl et al. (55)	Breast and colon	1	^90^Y-FAPI	n/a	n/a	4	PD
Jokar et al. (66)	Breast	1	^177^Lu-FAPI46	3.7 GBq (*per cycle*)	n/a	2	n/a
Assadi et al. (68)	Various	21	^177^Lu-FAPI46	3.7GBq (*mean*)	n/a	2 (*mean*)	SD (*n* = 12) and PD (*n* = 6)

SD, stable disease; PD, progressive disease; n/a, not available.

## Limitations

Although there is emerging FAP-targeted PET/CT data available for cancer imaging, activated fibroblasts, particularly in tissue-remodeling processes, can also express FAPs. As a result, circumstances such as the differentiation of chronic inflammatory or wound-healing processes and malignancy limit the specificity of FAPI PET imaging for certain cancer entities such as pancreatic cancer (52). On the other hand, this allows its use in non-oncological diseases; however, other than the case reports, FAPI PET imaging of nonmalignant conditions has focused on cardiovascular (69–71) and rheumatological (68, 72, 73) diseases. Eventually, clinical adoption of FAPI PET requires understanding the limitations of FAPI PET, its use in cancer-specific and non-oncological applications, which can be achieved in the long term.

## Conclusion

In diagnostic oncology, targeting increased glucose uptake as a hallmark of cancer-associated metabolic changes by ^18^FDG PET/CT is unrivaled. Its capability in detecting metabolic changes even in the absence of anatomical changes has led to high sensitivity; however, increased glycolysis is also common in various nonmalignant diseases and physiological processes, which causes low specificity. Several PET radiotracers have been developed in the last decades; however, they targeted specific cancer types. FAPIs have the potential to compete with FDG for diagnosis, staging, treatment planning, and therapy response assessment in many human solid tumors. In addition, it has the potential to be a theranostic modality for these tumors and likely transform the therapeutic options available, outside standard treatments, to millions of patients, in the future.

## Author Contributions

SK and YS: data collection and extraction and manuscript writing. RS: content planning, study design, and manuscript editing. All authors contributed to the article and approved the submitted version.

## Conflict of Interest

The authors declare that the research was conducted in the absence of any commercial or financial relationships that could be construed as a potential conflict of interest.

## Publisher’s Note

All claims expressed in this article are solely those of the authors and do not necessarily represent those of their affiliated organizations, or those of the publisher, the editors and the reviewers. Any product that may be evaluated in this article, or claim that may be made by its manufacturer, is not guaranteed or endorsed by the publisher.
